# Body Water Distribution and Phase Angle Are Differentially Related to Anaerobic Performance by Occupational Status in Firefighters: A Cross-Sectional Study

**DOI:** 10.3390/jfmk11040403

**Published:** 2026-10-03

**Authors:** Tiffany L. Adams, Michael R. Esco, Andrew T. LeMense, Samuel O. Gomez, Michael V. Fedewa, Changki Kim, Lee J. Winchester

**Affiliations:** 1 Tactical Performance Laboratory, Department of Kinesiology, The University of Alabama, Tuscaloosa, AL 35487, USA; tladams5@crimson.ua.edu (T.L.A.); sogomez@crimson.ua.edu (S.O.G.); ckim23@ua.edu (C.K.); 2 Department of Sport Science and Physical Education, Huntingdon College, Montgomery, AL 36106, USA; alemense@hawks.huntingdon.edu; 3 Body Composition Assessment Laboratory, Department of Kinesiology, The University of Alabama, Tuscaloosa, AL 35487, USA; mvfedewa@ua.edu

**Keywords:** bioimpedance analysis, cellular hydration, occupational physiology, body composition

## Abstract

**Background:** Bioimpedance-derived phase angle (PhA) and body water distribution may reflect cellular hydration and tissue quality, but their relevance to anaerobic performance in firefighters is unclear. **Objectives:** To compare these indices between firefighter recruits and incumbents and determine whether their relationships with anaerobic performance differed by occupational status. **Methods:** This retrospective cross-sectional study included recruits (*n* = 125) and incumbents (*n* = 71). Groups were compared for participant characteristics, bioimpedance variables, and Wingate performance. Pearson correlations and hierarchical regression tested relationships with relative peak (RelPP) and mean power (RelMP), including group × predictor interactions adjusted for fat mass (FM) and fat-free mass (FFM). Significance was set at *p* < 0.05. **Results:** Incumbents were older (38.89 ± 9.68 vs. 22.18 ± 4.47 years), heavier (95.14 ± 14.05 vs. 86.77 ± 13.72 kg), and had greater FM than recruits (*p* < 0.001). Recruits had higher RelPP and RelMP (*p* < 0.001), while body water distribution and PhA did not differ. In incumbents, intracellular water/total body water (ICW/TBW) and PhA were positively correlated with RelPP and RelMP, whereas extracellular water/total body water (ECW/TBW) was inversely correlated; these correlations were largely absent in recruits. After adjustment for FM and FFM, group × predictor interactions were observed for RelPP for PhA, ICW/TBW, ECW/TBW, and intracellular water/extracellular water (ICW/ECW) (*p* = 0.004–0.008), but not RelMP; these associations should be interpreted with caution given the number of comparisons examined. RelPP interactions remained significant after additional adjustment for age (*p* = 0.022–0.041). **Conclusions:** Despite similar body water distribution and PhA, recruits and incumbents differed in age, body mass, FM, and relative anaerobic performance. Occupational status appeared to moderate the relationships of these bioimpedance-derived indices with RelPP after adjustment for FM and FFM, with stronger relationships among incumbents.

## 1. Introduction

Firefighting is a physically demanding occupation requiring high levels of physical performance during critical tasks such as stair climbing, victim rescue, forcible entry, and equipment transport under time-sensitive conditions [1,2]. Although these activities require contributions from multiple energy systems, physiological responses during simulated firefighting demonstrate substantial aerobic demand together with considerable anaerobic involvement [1,3]. The intermittent nature of firefighting also requires repeated periods of high-intensity effort, placing substantial demands on anaerobic power during many critical occupational tasks [3,4]. Accordingly, anaerobic capacity has been identified as a key physiological determinant of firefighter occupational performance [5]. Therefore, identifying the physiological characteristics that influence anaerobic performance is of practical importance in this population, particularly those that may reflect underlying muscle quality and readiness for occupational tasks [5,6,7]. While previous research has largely focused on traditional body composition variables, including fat mass (FM) and fat-free mass (FFM) [8], these measures may not fully capture the functional properties of skeletal muscle that influence performance outcomes.

Bioelectrical impedance analysis (BIA) provides additional insight into tissue characteristics by quantifying body water distribution and cellular properties [9,10]. The relative distribution of intracellular water (ICW) and extracellular water (ECW) has been proposed as an indicator of muscle quality, as ICW is primarily associated with metabolically active tissue, whereas ECW reflects extracellular fluid and non-contractile components [9,11]. Ratios such as ICW to total body water (ICW/TBW), ECW to TBW (ECW/TBW), and ICW to ECW (ICW/ECW) therefore provide a means of assessing fluid compartmentalization beyond traditional body composition metrics [12,13]. A greater proportion of ICW relative to ECW may reflect a more favorable intracellular environment for force production and metabolic function, whereas a higher ECW proportion may indicate reduced muscle quality [13,14].

In addition to fluid distribution, phase angle (PhA), derived from BIA, has emerged as a marker of cellular integrity and function [15]. Mechanistically, PhA reflects the relationship between resistance and reactance, providing an indirect measure of cell membrane capacitance and intracellular structural integrity [16]. Higher PhA values have been associated with greater cell membrane integrity, increased body cell mass, and improved muscle function [15,17]. Thus, PhA may serve as a global indicator of cellular health that complements measures of body water distribution [16,17].

Despite the theoretical relevance of these bioimpedance-derived markers, their relationships with anaerobic performance remain unclear, particularly in occupational populations. It is also unknown whether these relationships are independent of traditional body composition variables such as FM and FFM, which are established contributors to anaerobic performance [18]. Furthermore, occupational status may influence these relationships [2]. Firefighter recruits are typically selected for entry into training programs and may exhibit less variability in physical fitness and physiological characteristics. In contrast, incumbent firefighters may exhibit greater variability due to differences in occupational exposure, aging, training habits, and lifestyle factors accumulated over time [19,20,21]. These differences may alter the physiological relevance of body water distribution and cellular integrity in relation to performance.

Therefore, the purpose of this study was to compare bioimpedance-derived markers of body water distribution (ICW/TBW, ECW/TBW, ICW/ECW) and phase angle between firefighter recruits and incumbents and determine whether their relationships with anaerobic performance differed by occupational status. A secondary aim was to evaluate whether these relationships were independent of FM and FFM. It was hypothesized that more favorable body water distribution (i.e., higher ICW relative to ECW) and higher phase angle would be associated with greater relative anaerobic performance, and that these relationships would be stronger in incumbent firefighters than in recruits, reflecting differences in physiological adaptation and variability associated with occupational experience.

## 2. Materials and Methods

### 2.1. Study Design

This retrospective cross-sectional study used physiological assessment data collected during routine occupational and training fitness evaluations of firefighter recruits and incumbent firefighters in the Southeastern United States. Assessments were conducted in occupational fitness testing environments by trained research personnel. The manuscript was reviewed in accordance with the Strengthening the Reporting of Observational Studies in Epidemiology (STROBE) checklist for cross-sectional studies. The primary objective was to compare bioimpedance-derived body water distribution variables and PhA between firefighter recruits and incumbents and determine whether their relationships with anaerobic performance differed by occupational status. A secondary objective was to evaluate whether these relationships were independent of FM and FFM.

### 2.2. Participants

**Figure 1:**
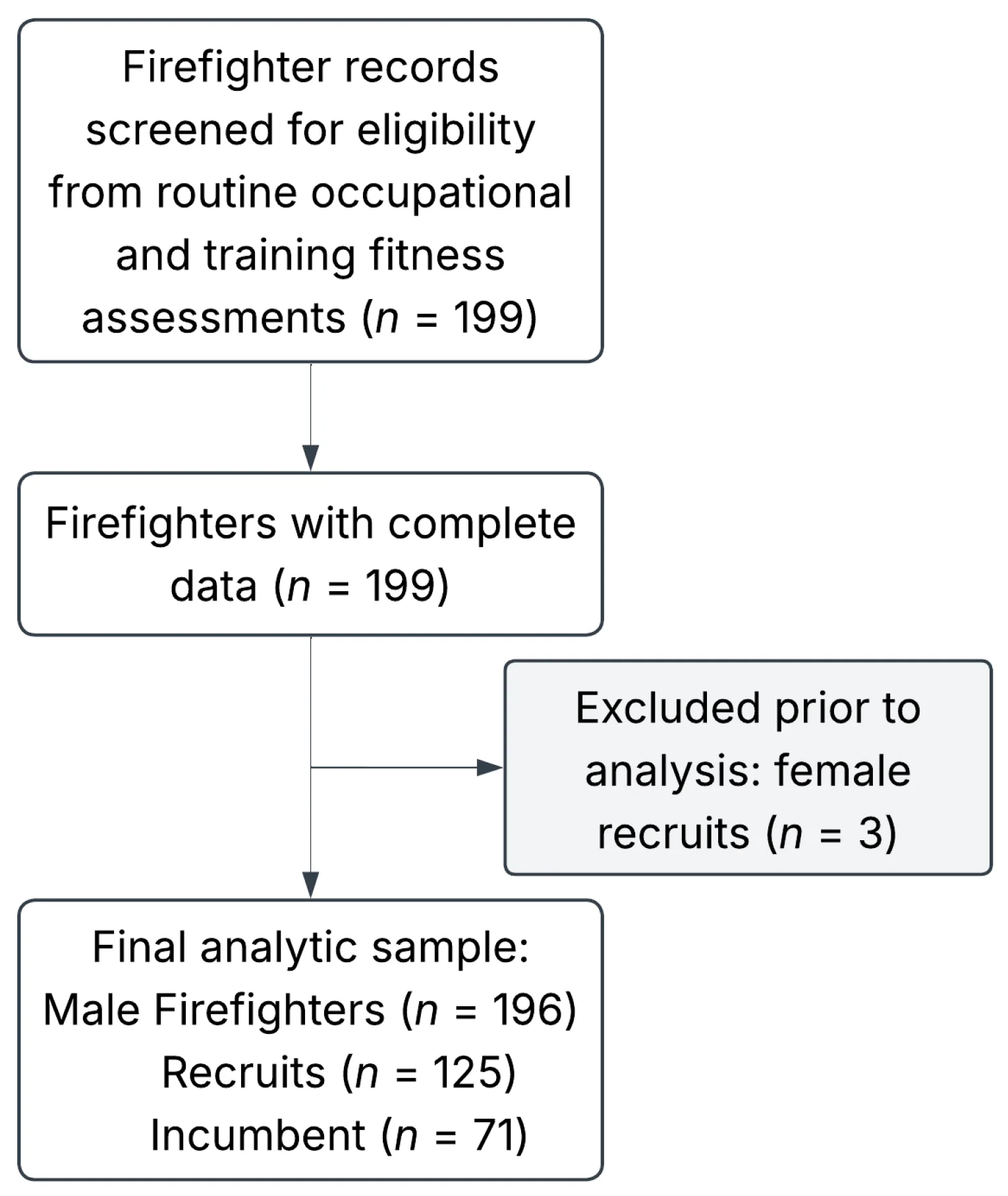
Flowchart for participant selection and inclusion criteria.

A total of 199 firefighter records were initially screened for eligibility from routine occupational and training fitness assessments conducted in the Southeastern United States. Three female recruit participants were excluded prior to analysis due to the very small number of female participants and the potential influence of sex-related differences in body composition and body water distribution. Therefore, the final analytic sample included 196 male firefighters, consisting of 125 recruits and 71 incumbent firefighters. Participant selection and inclusion in the final analytic sample are summarized in Figure 1. Because this study involved a retrospective analysis of existing occupational fitness assessment data, no a priori sample-size calculation was performed. The analytic sample comprised all records meeting the final inclusion criteria during the study period.

Recruits were individuals entering a structured regional firefighter training program, whereas incumbents were active professional firefighters who completed physical testing as part of their departmental fitness evaluations. Participants were classified by biological sex indicated by self-report on intake forms. All participants were free from known cardiovascular, metabolic, or musculoskeletal conditions that would preclude maximal exercise testing. Prior to participation, individuals were informed of testing procedures and provided written consent in accordance with institutional guidelines. All procedures were approved by the appropriate institutional review board (Protocol #23-08-6855).

### 2.3. Experimental Procedures

The order of testing for all participants was as follows: (1) anthropometric measurements, (2) body composition assessment, and (3) anaerobic performance assessment via the Wingate protocol. Participants received the testing facility’s standard pre-assessment instructions regarding hydration and preparation prior to evaluation. However, because the present study was retrospective, documentation sufficient to verify the exact pre-test fluid intake of each participant or adherence to these instructions during the preceding 12-h was not available. To minimize potential influences of acute physiological fluctuations on bioelectrical impedance measurements, participants were instructed to remove shoes and heavy clothing and to void their bladder prior to assessment when possible. Body composition testing was performed before exercise testing to avoid the acute effects of physical activity on body fluid distribution. Testing was conducted according to the facility’s routine procedures. However, detailed information regarding time since the previous work shift, pre-test rest duration, and exact time of day was not consistently available in the records.

### 2.4. Body Composition and Body Water Assessment

Upon entering the laboratory, body mass (to the nearest 0.1 kg) and barefoot standing height (to the nearest 0.1 cm) were measured using a calibrated digital scale and stadiometer, respectively (BSM, 270B, InBody, Cerrito, CA, USA). Body composition and body water distribution were assessed using a multi-frequency bioelectrical impedance analyzer with hand and foot electrodes (InBody 970, Cerrito, CA, USA). The device estimates body composition by passing a low-level electrical current through the body and measuring impedance characteristics, which differ between tissues due to variations in water and electrolyte content.

Participants stood barefoot on the device platform, properly aligned with the foot electrodes, and grasped the hand electrodes. All BIA measurements were obtained following the manufacturer’s recommended procedures, ensuring standardized placement for each participant. The analyzer provided device-derived estimates of total body water (TBW), intracellular water (ICW), and extracellular water (ECW) in liters. The use of multifrequency BIA for estimating body water compartments has been supported in previous validation studies [9,11,12,13,14].

In addition to absolute body water values, body water distribution ratios were calculated to characterize relative fluid distribution within tissues. These included ECW/TBW, ICW/TBW, and ICW/ECW. These ratios were included to provide indicators of tissue hydration characteristics and cellular fluid distribution that may reflect muscle quality and physiological status.

### 2.5. Anaerobic Performance Assessment

Anaerobic performance was assessed using a 30s Wingate anaerobic test performed on a mechanically braked cycle ergometer (Excalibur Sport, Lode, Groningen, The Netherlands). Prior to testing, participants completed a standardized warm-up consisting of five minutes of low-intensity cycling with a standardized revolution per minute (RPM) range (65–75 RPM).

Following the warm-up, participants were instructed to begin pedaling as fast as possible before the resistance load, 7.5% of the participant’s body mass, was applied for 30 s. Participants were verbally encouraged throughout the test to maintain maximal effort for the entire test duration. After 30 s, the resistance load was removed, and participants continued to pedal for a 5 min cool-down period.

Power output was continuously recorded by the ergometer software (LEM; Version 10; Lode BV, Groningen, The Netherlands), with the following performance variables being derived from the Wingate test: absolute peak power (W), relative peak power (W·kg^−1^), absolute mean power (W), relative mean power (W·kg^−1^), and fatigue rate. Relative performance metrics were calculated by normalizing power output to body mass.

### 2.6. Statistical Analysis

All statistical analyses were performed using IBM SPSS Statistics (Version 31.0; IBM Corp., Armonk, NY, USA). Descriptive statistics are presented as mean ± standard deviation (M ± SD). Normality was assessed using the Shapiro–Wilk test together with visual inspection of histograms and Q–Q plots. Although minor departures from normality were identified for some variables, visual inspection indicated no substantial violations of normality. Accordingly, the planned parametric analyses were retained.

Independent samples *t*-tests were used to compare demographic characteristics, body composition, body water variables, and Wingate performance metrics between firefighter recruits and incumbent firefighters. Effect sizes for between-group comparisons were quantified using Cohen’s d and are reported with 95% confidence intervals. Because 11 variables were compared using the independent-samples *t*-tests, Bonferroni-adjusted *p*-values were used to determine statistical significance and control the Type 1 error rate.

Exploratory Pearson product–moment correlation coefficients were calculated separately within each occupational group to examine relationships between body water variables, phase angle (PhA), and anaerobic performance outcomes. Differences in correlation coefficients between groups were evaluated using Fisher r-to-z transformations. Because these analyses were exploratory and used to justify the use of the interaction term in the hierarchical linear regression analysis, *p*-values were not adjusted. Hierarchical linear regression analysis was used to examine whether occupational status moderated the relationships between body water variables (ICW/TBW, ECW/TBW, ICW/ECW) and PhA with relative anaerobic performance (RelPP and RelMP). Prior to analysis, the continuous predictors (ICW/TBW, ECW/TBW, ICW/ECW, and PhA) were centered around their respective means, and occupational status was categorized as recruit or incumbent (coded as 0 and 1, respectively). Each model included the main effects of group and predictor and a Group × Predictor interaction term. FM and FFM were included as covariates in all models. Parameter estimates are presented as unstandardized B coefficients. Full models including the Group × Predictor interaction term were compared with reduced models containing the covariates and main effects using R^2^ and ΔR^2^. ΔR^2^ represents the incremental proportion of variance explained by the addition of the interaction term, with its statistical significance evaluated using the corresponding F-change test. Additional sensitivity analyses were conducted by including mean-centered age as a covariate, along with FM and FFM, in each moderation model to evaluate the robustness of the primary findings to age adjustment. Assumptions of linearity, normality, homoscedasticity, and absence of multicollinearity were assessed prior to analysis. Statistical significance was evaluated using an α = 0.05.

## 3. Results

Group comparisons between descriptive characteristics are presented in Table 1 and study variables are presented in Table 2. Incumbent firefighters were older and had greater body mass and FM than recruits. These observed ES ranged from −0.61 to −2.45 and indicated moderate to large differences between groups (*p* < 0.001). Large differences were observed in relative anaerobic performance and were higher in recruits for both peak and mean power (*p* < 0.001), whereas small, non-significant differences for absolute power outputs were observed between groups (*p* > 0.05). Small non-significant group differences were also observed for body water distribution variables (ICW/TBW, ECW/TBW, ICW/ECW; *p* > 0.05) or phase angle (*p* = 0.688).

Exploratory Pearson correlation coefficients describing relationships between body water ratios, phase angle (PhA), and relative anaerobic performance are presented in Table 3. In recruits, weak, non-significant associations between body water ratios, RelPP and RelMP were observed (*p* > 0.05). PhA demonstrated weak positive correlations with both RelPP (r = 0.18, *p* < 0.05) and RelMP (r = 0.20, *p* < 0.05). In contrast, several moderate correlations were observed in incumbent firefighters. ICW/TBW was positively correlated with RelPP (r = 0.52, *p* < 0.001) and RelMP (r = 0.42, *p* < 0.001), whereas ECW/TBW was inversely correlated with RelPP (r = −0.52, *p* < 0.001) and RelMP (r = −0.42, *p* < 0.001). Similar positive correlations were observed for ICW/ECW with RelPP (r = 0.52, *p* < 0.001) and RelMP (r = 0.42, *p* < 0.001). PhA demonstrated the strongest correlations among incumbents, with positive relationships observed for RelPP (r = 0.60, *p* < 0.001) and RelMP (r = 0.52, *p* < 0.001). Fisher r-to-z transformations indicated significantly stronger correlations among incumbents for all RelPP comparisons and for the PhA–RelMP relationship, whereas between-group differences for the RelMP correlations involving ICW/TBW, ECW/TBW, and ICW/ECW did not reach statistical significance (Table 3). Absolute body water measures (TBW, ICW, ECW) and absolute anaerobic performance variables were not significantly related in either group (all *p* > 0.05).

The hierarchical linear regression analyses examining occupational status as a moderator of the relationships between body water variables, phase angle, and relative anaerobic performance outcomes, while controlling for FM and FFM, are presented in Table 4. All models were significant (*p* < 0.001), explaining 47.2–52.1% of the variance (R^2^ = 0.472–0.521). For RelPP, significant Group × Predictor interaction effects were observed for all variables.

**Figure 2:**
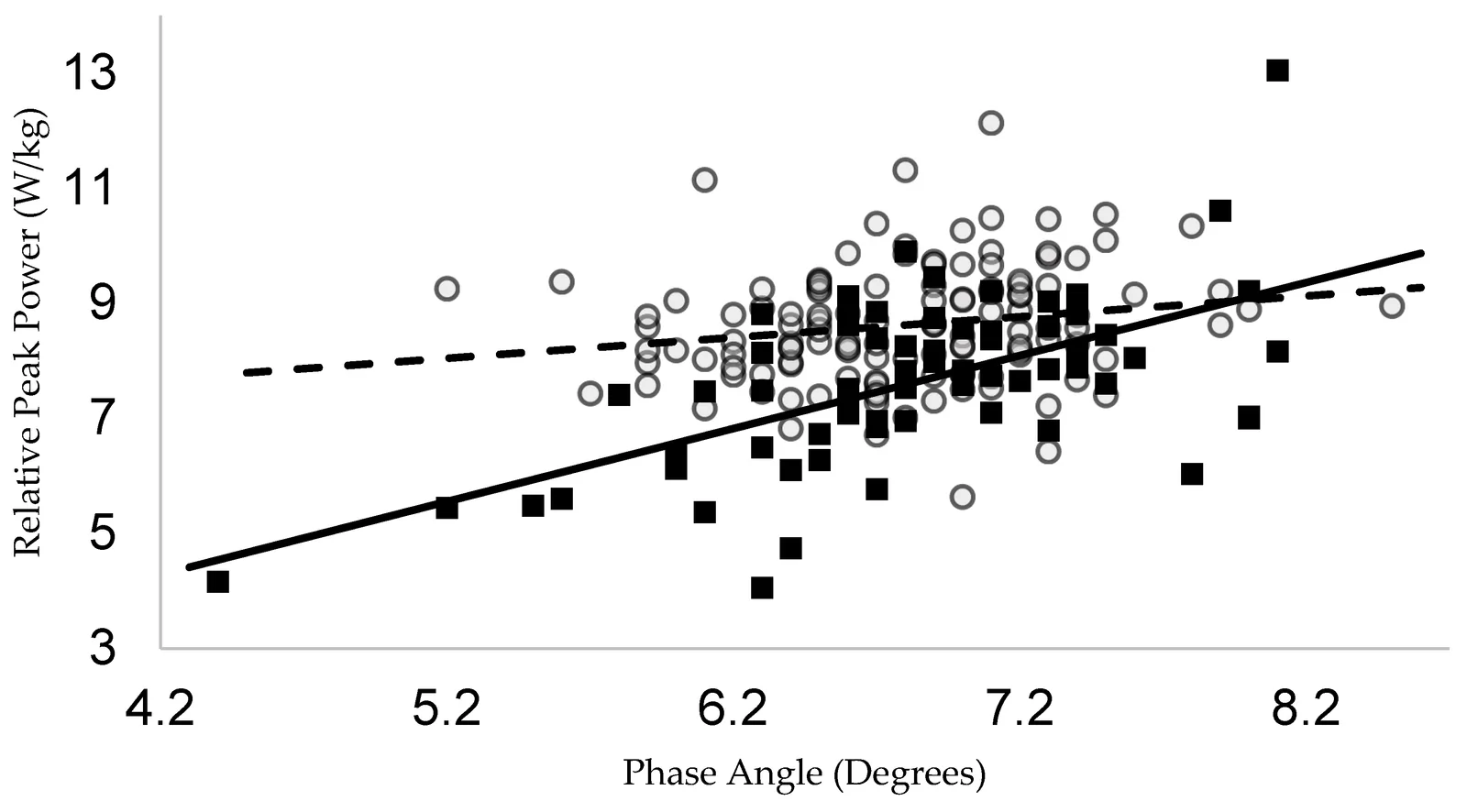
Relationship between phase angle (PhA) and relative peak power (RelPP) in firefighter recruits (*n* = 125) and incumbent firefighters (*n* = 71). A stronger positive association between PhA and RelPP is observed in incumbent firefighters compared to recruits, consistent with a significant Group × PhA interaction after controlling for fat mass and fat-free mass. Gray circles, Dashed line: Recruits; Black squares, Solid line: Incumbents.

PhA demonstrated a significant interaction (B = 0.618, ΔR^2^ = 0.019, *p* = 0.008, Figure 2), as did ICW/TBW (B = 64.789, ΔR^2^ = 0.023, *p* = 0.004), ECW/TBW (B = −64.101, ΔR^2^ = 0.022, *p* = 0.005), and ICW/ECW (B = 9.082, ΔR^2^ = 0.023, *p* = 0.004). In all models, FM was inversely associated with RelPP (*p* < 0.001), whereas FFM was positively associated with RelPP in models including ICW/TBW (*p* = 0.011), ECW/TBW (*p* = 0.011), and ICW/ECW (*p* = 0.011), but not phase angle (*p* = 0.239). These results indicate that lower FM and higher FFM were frequently associated with higher RelPP.

For RelMP, no significant interaction effects were observed (*p* > 0.05). Phase angle (B = 0.164, ΔR^2^ = 0.003, *p* = 0.317), ICW/TBW (B = 17.736, ΔR^2^ = 0.003, *p* = 0.267), ECW/TBW (B = −17.178, ΔR^2^ = 0.003, *p* = 0.282), and ICW/ECW (B = 2.479, ΔR^2^ = 0.003, *p* = 0.264) did not significantly interact with group. Similar to RelPP, FM was inversely associated with RelMP across all models (*p* < 0.001), whereas FFM was positively associated with RelMP in the ICW/TBW, ECW/TBW, and ICW/ECW models (*p* = 0.023), but not in the PhA model (*p* = 0.261).

Because age differed significantly between recruits and incumbent firefighters, additional sensitivity analyses were conducted with age included as a covariate along with FM and FFM. For RelPP, the Group × Predictor interactions remained significant for PhA (B = 0.504, 95% CI [0.021, 0.988], ΔR^2^ = 0.011, *p* = 0.041), ICW/TBW (B = 55.147, 95% CI [7.621, 102.674], ΔR^2^ = 0.015, *p* = 0.023), ECW/TBW (B = −54.549, 95% CI [−102.022, −7.077], ΔR^2^ = 0.014, *p* = 0.025), and ICW/ECW (B = 7.729, 95% CI [1.132, 14.327], ΔR^2^ = 0.015, *p* = 0.022). In contrast, no significant Group × Predictor interactions were observed for RelMP after adjustment for age (all *p* ≥ 0.664). Thus, additional adjustment for age did not alter the overall pattern of the moderation findings.

## 4. Discussion

The primary finding of this study was that bioimpedance-derived markers of body water distribution and PhA were more strongly associated with relative anaerobic performance in incumbent firefighters than in recruits, with occupational status moderating these relationships. Notably, these relationships were observed for relative performance measures and were most robust for relative peak power. Importantly, the Group × Predictor interactions for RelPP remained significant in sensitivity analyses that additionally adjusted for age, whereas the RelMP interactions remained nonsignificant. Collectively, these findings suggest that these bioimpedance-derived markers may function as context-dependent indicators of physiological function and performance capacity rather than universal determinants across populations.

Before examining the bioimpedance-specific findings, the overall group differences warrant consideration. Compared to recruits, incumbent firefighters were older, heavier, exhibited greater FM, and demonstrated lower relative anaerobic performance. These findings are not unexpected and likely reflect the cumulative effects of aging, occupational exposure, and less uniform training status among incumbent personnel [2,19,22]. In contrast, recruits are generally entering training from a more recently conditioned state and are often preparing specifically for academy-related physical demands. Therefore, the lower relative peak and mean power observed in incumbents may be partly explained by their greater body mass and adiposity, both of which would be expected to negatively influence power output when expressed relative to body mass [8,23].

The absence of group differences in body water distribution and PhA, despite clear differences in age, body composition, and relative anaerobic performance, presents an important paradox. These findings suggest that bioimpedance-derived markers of cellular hydration and integrity do not function as simple discriminators of occupational status, but rather as context-dependent indicators of performance capacity [16,24]. This finding highlights that the physiological meaning of these indices cannot be inferred from group-level comparisons alone. In this regard, the stronger and more consistent associations observed in incumbent firefighters suggest that the physiological relevance of these variables may become more apparent under conditions of greater heterogeneity in physical conditioning and physiological status.

One possible explanation for these group-specific relationships is greater heterogeneity in physiological and conditioning characteristics among incumbent firefighters. Recruits may represent a more uniformly conditioned group in several determinants of anaerobic performance, whereas incumbent firefighters may exhibit greater variability associated with differences in occupational experience, training practices, and lifestyle factors [19,20,21]. Such heterogeneity could increase the extent to which cellular-level characteristics differentiate performance outcomes. However, training practices, habitual physical activity, lifestyle behaviors, and related occupational exposures were not directly measured in the present study. Therefore, this explanation remains speculative and should be evaluated in future research.

Indeed, within incumbent firefighters, a higher proportion of ICW relative to ECW was associated with greater relative anaerobic performance, whereas a higher ECW proportion was associated with lower performance. These findings align with the conceptualization of ICW as a marker of metabolically active tissue and cellular function, while ECW may reflect non-contractile components or fluid distribution patterns less favorable for force production [13]. From a physiological perspective, a greater intracellular fluid environment may support enhanced metabolic activity, substrate utilization, and excitation–contraction processes within skeletal muscle, thereby contributing to improved power output when expressed relative to body mass [16,25,26,27]. In contrast, a higher proportion of extracellular water may be indicative of reduced muscle quality or altered fluid balance, which could negatively influence force transmission and mechanical efficiency [16].

Interestingly, PhA demonstrated the strongest associations with relative anaerobic performance in incumbents. Given that PhA is derived from the relationship between bioimpedance resistance and reactance, it is thought to reflect cell membrane capacitance and the functional status of intracellular structure [16,24,28]. In addition, PhA has been proposed as an integrative marker of cellular health, capturing multiple aspects of tissue quality including membrane integrity and fluid distribution [28]. The strong associations observed in the present study suggest that these combined cellular characteristics may be particularly relevant for rapid force production and high-power output. Accordingly, the present findings indicate that these relationships could potentially differ by occupational status, but they do not identify the physiological or behavioral mechanisms responsible for those differences.

An important observation is that the relationships between bioimpedance-derived variables and anaerobic performance were evident only when power output was expressed relative to body mass, with no significant associations observed for absolute metrics. Absolute power output is largely influenced by total muscle mass and body size [29,30]. In contrast, relative power reflects the ability to generate force in proportion to body mass, incorporating elements of neuromuscular efficiency, tissue quality, and metabolic function [29,31,32]. Thus, the observed associations with RelPP and RelMP indicate that intracellular fluid balance and cellular integrity may influence the functional capacity of existing muscle tissue rather than overall force production. This interpretation is consistent with the lack of association between absolute body water measures (e.g., total body water, ICW, ECW) and absolute performance outcomes, suggesting that the quantity of body water alone is not a primary determinant of anaerobic power. Instead, the distribution of fluid and the integrity of cellular structures appear to be more relevant for optimizing anaerobic performance relative to body mass. These results are consistent with the interpretation that these BIA-derived measures may relate to tissue-level properties beyond muscle quality alone; however, muscle quality itself was not directly assessed in this study, and this interpretation should be regarded as a possible physiological mechanism rather than a direct finding.

An additional noteworthy finding was that while occupational status moderated the relationships between bioimpedance-derived variables and relative anaerobic performance, the strength and independence of these relationships differed when accounting for body composition. At the bivariate level, significant associations were observed for both RelPP and RelMP in incumbent firefighters. However, after adjustment for FM and FFM, the interaction effects remained significant for RelPP but not for RelMP. This pattern was unchanged in the sensitivity analyses that additionally adjusted for age, with all four Group × Predictor interactions remaining significant for RelPP and all remaining nonsignificant for RelMP. These findings suggest that the relationships with RelPP were more robust to adjustment for both body composition and age, whereas the bivariate relationships observed for RelMP were not maintained in the multivariable moderation model.

From a physiological perspective, RelPP reflects the capacity for rapid force generation and is strongly influenced by neuromuscular function, rate of force development, and the immediate intracellular bioenergetic response [31,33,34]. These processes may be more directly linked to cellular-level properties such as intracellular fluid balance and membrane integrity [16,27], which may explain these significant relationships after accounting for body composition. In contrast, relative mean power represents sustained anaerobic output and is influenced by a broader range of factors, including metabolic efficiency, fatigue resistance, and glycolytic energy system contribution [35,36]. The loss of significant interaction effects for RelMP after adjustment for FM and FFM suggests that these relationships were less independent of overall body composition than those observed for RelPP. Notably, FM was consistently inversely associated with RelMP across all models, supporting a potentially important role of adiposity in relative mean power performance [37,38].

Given the substantial age differences between recruits and incumbent firefighters, it is possible that the observed moderation effects may be influenced, in part, by age rather than occupational status. In addition, age-related declines in anaerobic performance and alterations in cellular fluid distribution and integrity have been previously reported [39,40,41]. Therefore, age was evaluated in additional sensitivity analyses. After age was added as a covariate along with FM and FFM, the Group × Predictor interactions for RelPP remained significant across PhA, ICW/TBW, ECW/TBW, and ICW/ECW, although the magnitude of the interaction effects was attenuated. In contrast, the Group × Predictor interactions for RelMP remained nonsignificant. These findings indicate that the primary moderation pattern was not eliminated by adjustment for age. Nevertheless, because age and occupational status are inherently related in this cohort, their independent contributions cannot be fully disentangled in the present cross-sectional design. Accordingly, the observed moderation effects should be interpreted as differences between occupational groups rather than as effects attributable exclusively to occupational status independent of age.

Several limitations should be acknowledged. The retrospective, cross-sectional design precludes causal inference, and longitudinal research is needed to determine whether changes in body water distribution or phase angle are associated with changes in performance. Between-group differences were corrected for multiple comparisons (e.g., via Bonferroni adjustment). However, the exploratory correlation analysis was used to justify the hierarchical linear regression model and interaction term, and therefore, *p*-values were not adjusted. The use of R^2^ and ΔR^2^, as well as the individual beta coefficients and corresponding *p*-values, addresses the contribution of each independent variable while accounting for the other variables in the model. These findings should be interpreted as exploratory and hypothesis-generating rather than confirmatory. However, the use of R^2^ and ∆R^2^, as well as the individual beta coefficients (and corresponding *p*-values) answered different questions about the relative importance of the independent variables conditional on the other independent variables included in each model. As such, these results do not seek to compare which measure of TBW was more important than the others, but rather indicate that the relative importance of FM and FFM was comparable across all models regardless of how TBW was expressed. The ECW and ICW variables are related to each other, as if water is not intracellular, it is extracellular, and vice versa. Including each variable in the same model would result in significant collinearity and overlap between the measures of TBW when predicting anaerobic performance. Perhaps the most important findings are that TBW, whether measured as ICW/TBW, ECW/TBW, or the ratio of ICW/ECW, is associated with anaerobic performance, and the relative contributions (beta coefficients and *p* values) for group, FM, and FFM are comparable across each of the models. Additionally, bioelectrical impedance analysis provides indirect estimates of body water compartments and cellular properties, which may be influenced by hydration status and measurement conditions. In addition, independent criterion validation specifically for the ICW and ECW estimates generated by the InBody 970 remains limited. Although standardized pre-test procedures were used at the testing facility, individual adherence to hydration instructions and detailed information regarding shift-related fatigue, pre-test rest, and time of day could not be verified retrospectively. Differences in age between recruits and incumbent firefighters also represent an important limitation. Although the primary moderation findings remained significant for RelPP after additional adjustment for age, the close relationship between age and occupational status in this cohort limits the ability to isolate their independent contributions. Future research should examine these relationships in age-matched cohorts or longitudinal designs that more directly separate aging from occupational experience. Furthermore, the use of distinct occupational groups, combined with the lack of detailed information regarding habitual physical activity and training practices, may limit the ability to fully determine the effects of training status, lifestyle behaviors, and occupational exposure. Future studies should aim to more directly quantify these factors to better understand their role in moderating the relationship between cellular hydration, integrity, and anaerobic performance.

## 5. Conclusions

In conclusion, body water distribution indices and PhA did not differ between firefighter recruits and incumbents. However, their relationships with anaerobic performance differed by occupational status, with stronger relationships generally observed among incumbent firefighters. Moderation analyses further identified significant Group × Predictor interactions for RelPP across PhA, ICW/TBW, ECW/TBW, and ICW/ECW after adjustment for FM and FFM, whereas no significant moderation effects were observed for RelMP. The RelPP moderation pattern also remained significant after additional adjustment for age. These findings indicate that bioimpedance-derived measures of body water distribution and PhA may have population-specific relevance to peak anaerobic performance rather than serving as universal indicators across firefighter groups. These findings should be interpreted as exploratory and should be confirmed in future studies with age-matched cohorts.

## Figures and Tables

**Table 1:** Descriptive characteristics of firefighter recruits and incumbent firefighters. Values are presented as mean ± standard deviation.

Variable	Recruits (*n* = 125)	Incumbents (*n* = 71)	*p*-Value	Cohen’s d [95%CI]
Age (years)	22.18 ± 4.47	38.89 ± 9.68	<0.001 *	−2.45 [−2.83, −2.07]
Height (cm)	179.6 ± 6.84	180.3 ± 5.78	0.434	−0.12 [−0.41, 0.18]
Body Mass (kg)	86.77 ± 13.72	95.14 ± 14.05	<0.001 *	−0.61 [−0.90, −0.31]
Fat Mass (kg)	16.09 ± 6.83	22.08 ± 8.82	<0.001 *	−0.79 [−1.09, −0.49]
Fat-Free Mass (kg)	70.49 ± 9.21	72.87 ± 9.18	0.086	−0.26 [−0.55, 0.04]

cm = centimeters, kg = kilograms, * Indicates significance *p* < 0.05.

**Table 2:** Power and body water characteristics of firefighter recruits and incumbent firefighters. Values are presented as mean ± standard deviation.

Variable	Recruits (*n* = 125)	Incumbents (*n* = 71)	*p*-Value	Cohen’s d [95%CI]
Peak Power (W)	742.94 ± 117.64	720.56 ± 152.85	0.253	0.17 [−0.12, 0.46]
Mean Power (W)	536.81 ± 83.50	518.83 ± 98.40	0.169	0.21 [−0.09, 0.50]
Relative Peak Power (W·kg^−1^)	8.62 ± 1.03	7.63 ± 1.45	<0.001 *	0.83 [0.52, 1.13]
Relative Mean Power (W·kg^−1^)	6.25 ± 0.82	5.50 ± 0.96	<0.001 *	0.86 [0.55, 1.16]
Total Body Water (L)	51.65 ± 6.71	53.39 ± 6.73	0.083	−0.26 [−0.55, 0.03]
Intracellular Water (L)	32.48 ± 4.22	33.51 ± 4.18	0.099	−0.25 [−0.54, 0.05]
Extracellular Water (L)	19.17 ± 2.52	19.87 ± 2.59	0.065	−0.28 [−0.57, 0.02]
ICW/TBW	0.629 ± 0.005	0.628 ± 0.007	0.256	0.17 [−0.12, 0.46]
ECW/TBW	0.371 ± 0.005	0.372 ± 0.007	0.285	−0.16 [−0.45, 0.13]
ICW/ECW	1.695 ± 0.038	1.688 ± 0.052	0.302	0.15 [−0.14, 0.45]
Phase Angle (°)	6.80 ± 0.53	6.84 ± 0.68	0.688	−0.07 [−0.35, 0.23]

ICW/TBW = Intracellular water to total body water ratio, ECW/TBW = Extracellular water to total body water ratio, ICW/ECW = Intracellular water to extracellular water ratio, kg = kilograms, W = Watts, W·kg^−1^ = Watts per kilogram of body weight, L = liters, ° = degrees. * Indicates significance *p* < 0.05.

**Table 3:** Pearson correlation coefficients (r) describing relationships of body water variables and phase angle with anaerobic performance outcomes in recruits (*n* = 125) and incumbent firefighters (*n* = 71).

Variable	Recruits RelPP	Incumbents RelPP	Δr (z-test)	Recruits RelMP	Incumbents RelMP	Δr (z-test)
**ICW/TBW**	0.12	0.52 ^‡^	3.01 ^†^	0.15	0.42 ^‡^	1.96
**ECW/TBW**	−0.13	−0.52 ^‡^	−2.94 ^†^	−0.16	−0.42 ^‡^	−1.89
**ICW/ECW**	0.12	0.52 ^‡^	3.01 ^†^	0.16	0.42 ^‡^	1.89
**PhA**	0.18 *	0.60 ^‡^	3.38 ^‡^	0.20 *	0.52 ^‡^	2.47 *

RelPP = Relative peak power, RelMP = Relative mean power, ICW/TBW = Intracellular water to total body water ratio, ECW/TBW = Extracellular water to total body water ratio, ICW/ECW = Intracellular water to extracellular water ratio, PhA = Phase angle, Δr (z-test) = the Fisher r-to-z transformation comparing correlation coefficients between groups. * = *p* < 0.05, ^†^ = *p* < 0.01, ^‡^ = *p* < 0.001 for both Pearson correlations and Fisher r-to-z tests.

**Table 4:** Adjusted linear regression models examining occupational status as a moderator of the relationships between phase angle and body water variables with relative anaerobic performance outcomes after controlling for fat mass and fat-free mass.

Outcome	Predictor	R^2^	Group	Predictor	Group × Predictor	FM	FFM
RelPP	PhA	0.485	−0.589 (*p* < 0.001)	0.355 (*p* = 0.030)	0.618 (*p* = 0.008)	−0.034 (*p* < 0.001)	0.004 (*p* = 0.239)
	ICW/TBW	0.472	−0.480 (*p* = 0.002)	21.069 (*p* = 0.193)	64.789 (*p* = 0.004)	−0.037 (*p* < 0.001)	0.009 (*p* = 0.011)
	ECW/TBW	0.473	−0.463 (*p* = 0.002)	−22.441 (*p* = 0.165)	−64.101 (*p* = 0.005)	−0.037 (*p* < 0.001)	0.009 (*p* = 0.011)
	ICW/ECW	0.473	−0.486 (*p* = 0.001)	3.001 (*p* = 0.178)	9.082 (*p* = 0.004)	−0.037 (*p* < 0.001)	0.009 (*p* = 0.011)
RelMP	PhA	0.521	−0.375 (*p* < 0.001)	0.314 (*p* = 0.006)	0.164 (*p* = 0.317)	−0.030 (*p* < 0.001)	0.003 (*p* = 0.261)
	ICW/TBW	0.505	−0.318 (*p* = 0.003)	21.617 (*p* = 0.060)	17.736 (*p* = 0.267)	−0.032 (*p* < 0.001)	0.006 (*p* = 0.023)
	ECW/TBW	0.506	−0.319 (*p* = 0.003)	−22.509 (*p* = 0.049)	−17.178 (*p* = 0.282)	−0.032 (*p* < 0.001)	0.006 (*p* = 0.023)
	ICW/ECW	0.505	−0.321 (*p* = 0.003)	3.050 (*p* = 0.053)	2.479 (*p* = 0.264)	−0.032 (*p* < 0.001)	0.006 (*p* = 0.023)

RelPP = Relative peak power, RelMP = Relative mean power, ICW/TBW = Intracellular water to total body water ratio, ECW/TBW = Extracellular water to total body water ratio, ICW/ECW = Intracellular water to extracellular water ratio, PhA = Phase angle, FM = fat mass, FFM = fat-free mass.

## Data Availability

The data are not publicly available due to privacy and confidentiality considerations but may be available from the corresponding author upon reasonable request.

## Abbreviations

The following abbreviations are used in this manuscript:

BIA	Bioelectrical Impedance Analysis
ECW	Extracellular Water
ECW/TBW	Extracellular Water to Total Body Water Ratio
FFM	Fat-free Mass
FM	Fat Mass
ICW	Intracellular Water
ICW/ECW	Intracellular Water to Extracellular Water Ratio
ICW/TBW	Intracellular Water to Total Body Water Ratio
PhA	Phase Angle
RelMP	Relative Mean Power
RelPP	Relative Peak Power
RPM	Revolutions Per Minute
TBW	Total Body Water
